# Association between hysterectomy for benign indications and the risk of breast cancer: a systematic review and meta- analysis

**DOI:** 10.3389/fonc.2025.1600459

**Published:** 2025-11-20

**Authors:** Wenjing Liu, Tingting Lu, Jingqi Yang, Jie Liu

**Affiliations:** 1 Guang’anmen Hospital, China Academy of Chinese Medical Sciences, Beijing, China; 2 Institute of Basic Research in Clinical Medicine, China Academy of Chinese Medical Sciences, Beijing, China; 3 Graduate School, Beijing University of Chinese Medicine, Beijing, China

**Keywords:** hysterectomy, breast cancer, risk, meta-analysis, systematic review

## Abstract

**Objectives:**

To determine the association between hysterectomy performed for benign indications and the risk of developing BC.

**Methods:**

A literature search was conducted in PubMed, Embase, and the Cochrane Library from database inception up to December 11, 2024. Eligible studies were observational design. Relative ratios (RRs) and 95% confidence intervals (CIs) were pooled using a random-effects model, *I^2^* was used to assess the heterogeneity between studies.

**Results:**

This meta-analysis included 12 studies, consisting of 4 case-control studies and 8 cohort studies. The pooled analysis of case-control studies indicated that hysterectomy reduced the risk of BC (RR = 0.839, 95% CI: 0.707-0.995, *P* = 0.043, *I^2^* = 81.661%). However, the pooled analysis of cohort studies did not observe a significant association between hysterectomy and the occurrence of BC (RR = 0.981, 95% CI: 0.927-1.037, *P* = 0.495, *I^2^* = 60.319%).

**Conclusions:**

The present study reveals a protective effect of hysterectomy on the occurrence of BC in case-control studies. However, more studies, especially cohort studies, are needed to elucidate the potential beneficial effects of hysterectomy on the development of BC.

**Systematic Review Registration:**

https://www.crd.york.ac.uk/PROSPERO/view/CRD42024596235, identifier CRD42024612164.

## 1 Introduction

Breast cancer (BC) is the most common type of cancer among women worldwide and the leading cause of cancer-related deaths. In 2022, there were approximately 2.3 million new cases of BC globally, with over 660,000 deaths (1). According to the latest statistics from the United States, the incidence of BC is expected to continue rising in the future, with a noticeable trend of affecting younger individuals (2). Although the emergence of new therapies represented by targeted therapy and immunotherapy in recent years has greatly improved patients’ prognosis (3), these treatments not only require precise molecular typing and entail the inevitable issue of drug resistance but also carry a higher risk of toxicity and adverse reactions (4). Therefore, it is particularly important to accurately identify high-risk groups and factors for BC and scientifically carry out prevention and screening measures. Several studies have demonstrated that the occurrence and progression of BC are closely associated with genetic, hormonal, lifestyle, and environmental factors (5–7, 12), primarily involving body weight, diet, physical activity, alcohol consumption, reproductive characteristics, and BRCA gene mutations (8–11).

Hysterectomy is one of the most common gynecologic surgeries around the world (13). According to statistical reports from 2006, approximately 153,000 hysterectomies were performed in Germany that year (14), while the annual number of such procedures in the United States was about 600,000 (15). As of 2023, the prevalence of hysterectomy in the United States remains at 21.1% (16). Most hysterectomy procedures are used to treat symptomatic benign gynecologic conditions (such as uterine fibroids, endometriosis, and dysfunctional uterine bleeding) (17, 18). Hysterectomy for these benign indications can cause significant changes in hormone levels, which may affect the risk of hormone-related cancer (19). Some studies focus on hysterectomy combined with ovariectomy (20–22), which confirmed that hysterectomy combined with bilateral fallopian ovariectomy can reduce the risk of BC (23). However, the impact of simple hysterectomy on the occurrence rate of BC remains controversial. Some studies have found no association between the procedure and BC risk (24–30), while others have indicated that surgery performed before the age of 45 may reduce risk (20, 21, 31, 32). Furthermore, a retrospective study reported an increased occurrence rate of BC after surgery among women under 60 years of age, whereas a decreased rate was observed in those aged 60 and above (22).

Therefore, this systematic review and meta-analysis was conducted of the available evidence, aiming to investigate the association between hysterectomy and BC risk and explore the potential impact of hysterectomy on BC.

## 2 Methods

The study was conducted in accordance with the Meta-analysis of Observational Studies in Epidemiology (MOOSE) and reported in accordance with the Preferred Reporting Items for Systematic Reviews and Meta-Analyses (PRISMA) (33, 34). The study protocol was prospectively registered in PROSPERO (No.CRD42024612164) (35).

### 2.1 Search strategy

A comprehensive search was performed across PubMed, Embase, and Cochrane Library from database inception through November 11, 2024, using medical subject headings (MeSH) and free text words. The main search terms were used as follows: such as: (“Breast Neoplasms”[Mesh] OR “Breast Cancer” OR “Mammary Neoplasms”) AND (“Hysterectomy”[Mesh]) AND (“Risk Assessment” OR “Epidemiology”). Reference list was screened for eligible studies. Detailed search strategies are provided in **Supplementary Table S1-S3**.

### 2.2 Eligibility criteria

Eligible studies were required to fulfill these criteria: 1) Studies involving human participants without a history of BC prior to hysterectomy; 2) Studies where hysterectomy serve as the primary exposure and the incidence of BC as the outcome; 3) Studies providing one of the following metrics: Risk Ratio (RR), Hazard Ratio (HR), or Odds Ratio (OR) with 95% confidence intervals (CIs) to assess BC risk in patients after hysterectomy. 4) Observational design (cohort studies or case-control studies). For overlapping populations, we prioritized studies with larger sample sizes, longer follow-up durations, or more recent publication dates.

### 2.3 Exclusion criteria

Exclusion criteria comprised: 1) Non-original research (reviews, commentaries, conference abstracts); 2) Insufficient outcome data for effect size calculation; 3) Duplicate literatures.

### 2.4 Study selection

Independent reviewers (LWJ, LTT) conducted the study selection. After removing duplicates, the remaining records were screened by title/abstract and then full text of eligible articles. Any discrepancies were resolved through consulting with a third investigator (YJQ).

### 2.5 Data extraction

Data extraction was conducted independently by two reviewers (LWJ, LTT) using structured data collection forms. First author, publication year, country, study design, sample size, study period, exposure assessment method, follow-up duration, participant demographics, outcome measures, and metric were extracted. Discrepancies were resolved through consulting with YJQ.

### 2.6 Quality assessment

Methodological quality was independently evaluated by the two reviewers(LWJ, LTT) using the Newcastle-Ottawa Scale (NOS) (36). The NOS assesses three domains: selection process, group comparability, exposure (case–control study); or outcome (cohort study). Scores of 0-3, 4-6, and 7–9 were considered as low, moderate, and high quality, respectively.

### 2.7 Data synthesis and analysis

We conducted a meta-analysis based on different study types, such as case-control studies and cohort studies, rather than calculating an overall pooled effect estimate. This approach was taken to address the heterogeneity arising from differences in study design. RR was used as the common measure of association across studies, and HR and OR were considered approximate to RR given the low incidence rate of BC (37). Therefore, we converted the outcome metric (HR, and OR) into RR. Heterogeneity was evaluated by *I^2^* value. *I^2^* < 50% was considered as low heterogeneity, the fixed effect model was adopted. Otherwise, the random effect model was adopted. Sensitivity analyses evaluated result stability through sequential study exclusion. Publication bias was assessed by funnel plot symmetry and Egger’s test. Subgroup analyses were conducted according to age strata, follow-up duration, BC subtypes, and geographical regions. Statistical analysis was conducted using R 4.3.0 software. *P* value < 0.05 (bilateral) is considered significant.

## 3 Results

### 3.1 Literature search

**Figure 1:**
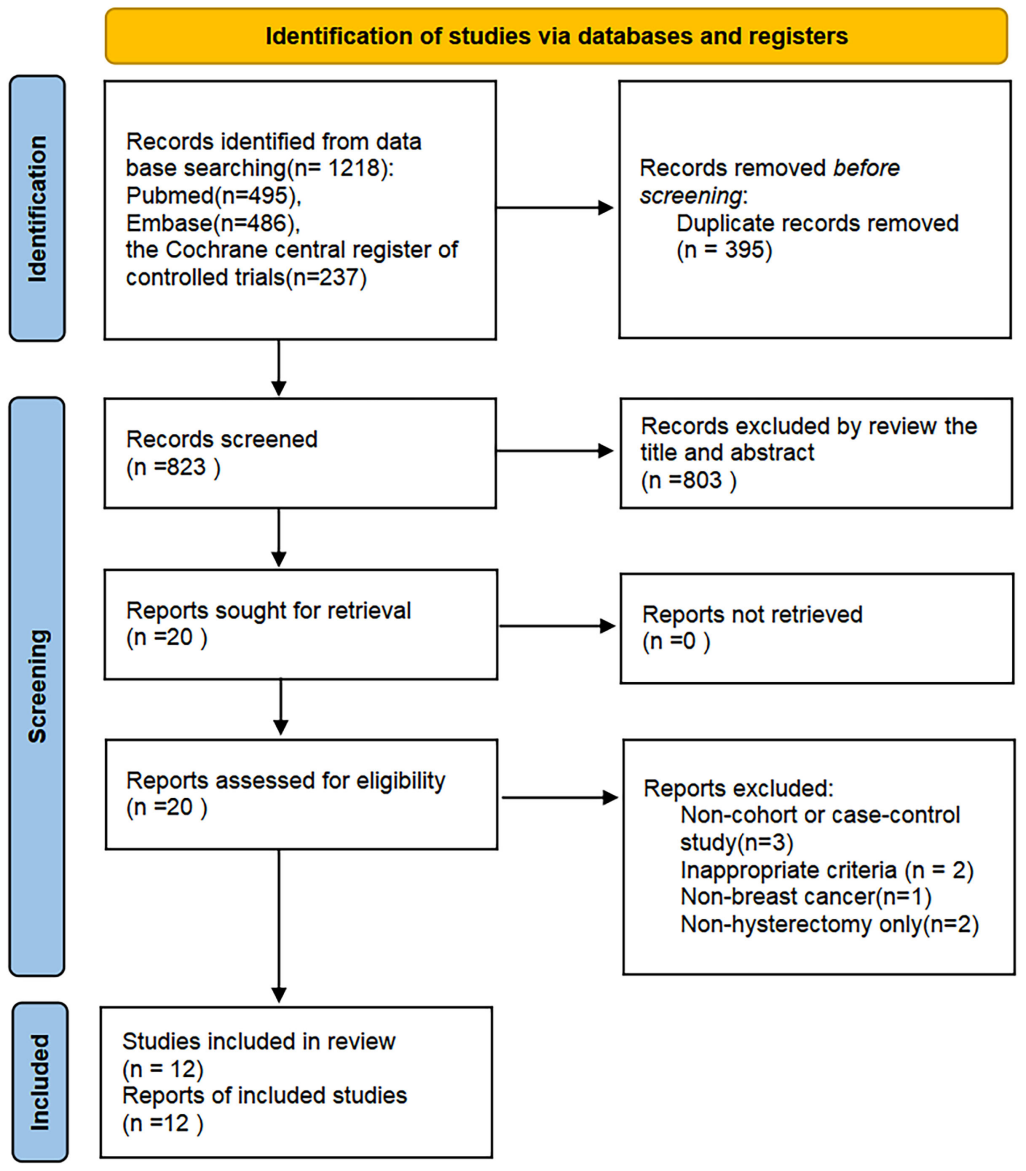
PRISMA flow diagram representing the reasons for exclusion.

The literature search initially identified 1,218 relevant records. After eliminating 395 duplicates, 823 records remained for the title and abstract screening. 20 articles needed to be read in full-text to determine their eligibility for inclusion. Then, eight studies were excluded for the following reasons: non-observational designs (n=3), inappropriate criteria (n=2), non-BC(n=1), non-hysterectomy only(n=3), with one additional study identified through supplementary citation tracking. Twelve rigorously conducted studies fulfilling all inclusion parameters were ultimately selected for meta-analytic integration. The complete study selection workflow is detailed in **Figure 1**.

### 3.2 Study characteristics

A total of 228313 participants were included in 12 studies between the years of 1997–2023.And the postoperative follow-up periods ranged from 0 to 27 years. Two studies were retrospective cohort studies (24, 32), 6 studies were prospective cohort studies (26, 27, 29–31, 38), and 4 studies were case-control studies (20, 21, 25, 28). Geographically, the majority originated from the United States (n=8), supplemented by single contributions from Australia, Sweden, Italy, and Finland. Detailed characteristics of included studies were summarized in **Table 1** and **Supplementary Table S3**.

**Table 1:** Characteristics of studies included in the systematic review and meta-analysis.

Study id	Country	Study design	Sample (n)	Study period	Follow-up years	Age	Exposure	Exposure ascertainment	Comparison	Outcomes	Outcomes ascertainment	Adjustments
Lovett et al., 2023 (38)	United States	Prospective cohort	6980	2003-2009	11.4	35-74	Hysterectomy	Questionnaire, self-report	Hysterectomy vs. No Hysterectomy	Breast cancer; n=601cases	Questionnaire, self-report	Race and ethnicity, family history of breast cancer, body mass index) or the breast cancer subtype of interest
Wilson et al., 2021 (32)	Australia	Retrospective cohort study	74056	1988-2014	27	Over 18	Hysterectomy	Cancer Register	Hysterectomy vs. No Hysterectomy	Breast cancer; n=2977 cases	ICD code	Age at entry, parity, remoteness category, SEIFA quintile, hospital-diagnosed fibroids, endometriosis, and prolapse
Altman et al., 2016 (30)	Sweden	Prospective cohort	90235	1973-2009	11.1	50.4	Hysterectomy	Swedish Inpatient Register	Hysterectomy vs. No Hysterectomy	Breast cancer; n=2201 cases	ICD code	Age, calendar years and parity
Robinson et al., 2016 (21)	United States	Case-control study	207 cases and 234 controls	1993-2001	/	20-74	Hysterectomy	North Carolina Cancer Registry	Hysterectomy vs. No Hysterectomy	Breast cancer	Diagnosed	Age, squared age, race, family history of breast cancer, alcohol consumption, age at menarche, parity and age at first pregnancy composite, lactation history, educational level, and smoking
Gaudet et al., 2014 (31)	United States	Prospective cohort	9655	1982-2001	13.9	50-74	Hysterectomy	The American Cancer Society	Hysterectomy vs. No Hysterectomy	Breast cancer; n=419 cases	Questionnaire, self-report	Age, race, education, alcohol consumption, smoking, parity, age at first birth, use of hormone replacement therapy, physical activity, age at menopause, and BMI
Boggs et al., 2014 (29)	United States	Prospective cohort	4756	1995-2011	16	21-69	Hysterectomy	Cancer Register	Hysterectomy vs. No Hysterectomy	Breast cancer; n=180 cases	ICD code	Age, BMI, menopausal hormone use, smoking status, educational attainment, geographic region, and family history
Nichols et al., 2012 (28)	United States	Case-control study	991 cases and 1106 controls	1992-2007	/	50-79	Hysterectomy	Cancer Register	Hysterectomy vs. No Hysterectomy	Breast cancer	Diagnosed	Age, study enrollment years, study site, age at menarche, age at first birth, parity, postmenopausal hormone use, body mass index, education, mammography screening, and family history
Press et al., 2011 (20)	United States	Case-control study	490 cases and 539 controls	1994-1998	/	35-64	Hysterectomy	The Centers for Disease Control and Prevention	Hysterectomy vs. No Hysterectomy	Breast cancer	Questionnaire	Age, race, study site, age at menarche, first-degree family history of breast cancer, number of term pregnancies, educational status, and duration of hormone therapy use
Jacoby et al., 2011 (27)	United States	Prospective cohort	11194	1993-1998	7.6	50-70	Hysterectomy	Clinical centers	Hysterectomy vs. No Hysterectomy	Breast cancer; n=309 cases	Questionnaire	Age, race/ethnicity, educational level, medical insurance, current health care provider, parity, body mass index, HT use and duration of use, and HT type.
Woolcott et al., 2009 (26)	United States	Prospective cohort	12785	1993-1996	7.7	45-75	Hysterectomy	Drivers' license records;Voter registration lists ;Health Care Financing Administration files	Hysterectomy vs. No Hysterectomy	Breast cancer; n=344 cases	Questionnaire, self-report	Age, BMI, family history, education, alcohol consumption, age at menarche, age at first birth, number of children, duration of current estrogen with progestin use, duration of current estrogen only use, and duration of past estrogen with progestin use
Parazzini et al., 1997 (25)	Italy	Case-control study	235 cases and 299 controls	1983-1994	/	20-74	Hysterectomy	Hospitals	Hysterectomy vs. No Hysterectomy	Breast cancer	Diagnosed	Terms for study, calendar year at interview, center, age, education, parity/age at first birth and family history
Luoto et al., 1997 (24)	Finland	Retrospective cohort study	18652	1967-1993	20.5	35-50	Hysterectomy	Finnish Cancer Registry	Hysterectomy vs. No Hysterectomy	Breast cancer; n=577 cases	ICD code	Education, parity, and follow-up.

ICD, the International Classification of Diseases.

### 3.3 Quality assessment

Using the NOS evaluation criteria, 3 studies were classified as nine-star (20, 25, 32), 1 study received eight stars (31), and 4 studies achieved seven-star ratings (21, 28–30), demonstrating high methodological quality. Four studies were categorized as moderate quality (six-star rating) (24, 26, 27, 38). The mean NOS score across all 12 publications was 7.25, suggesting an acceptable overall methodological standard. A comprehensive summary of the quality assessment process and outcomes is provided in **Supplementary Tables S2, S5**.

### 3.4 Meta-analysis

#### 3.4.1 Risk of BC

**Figure 2:**
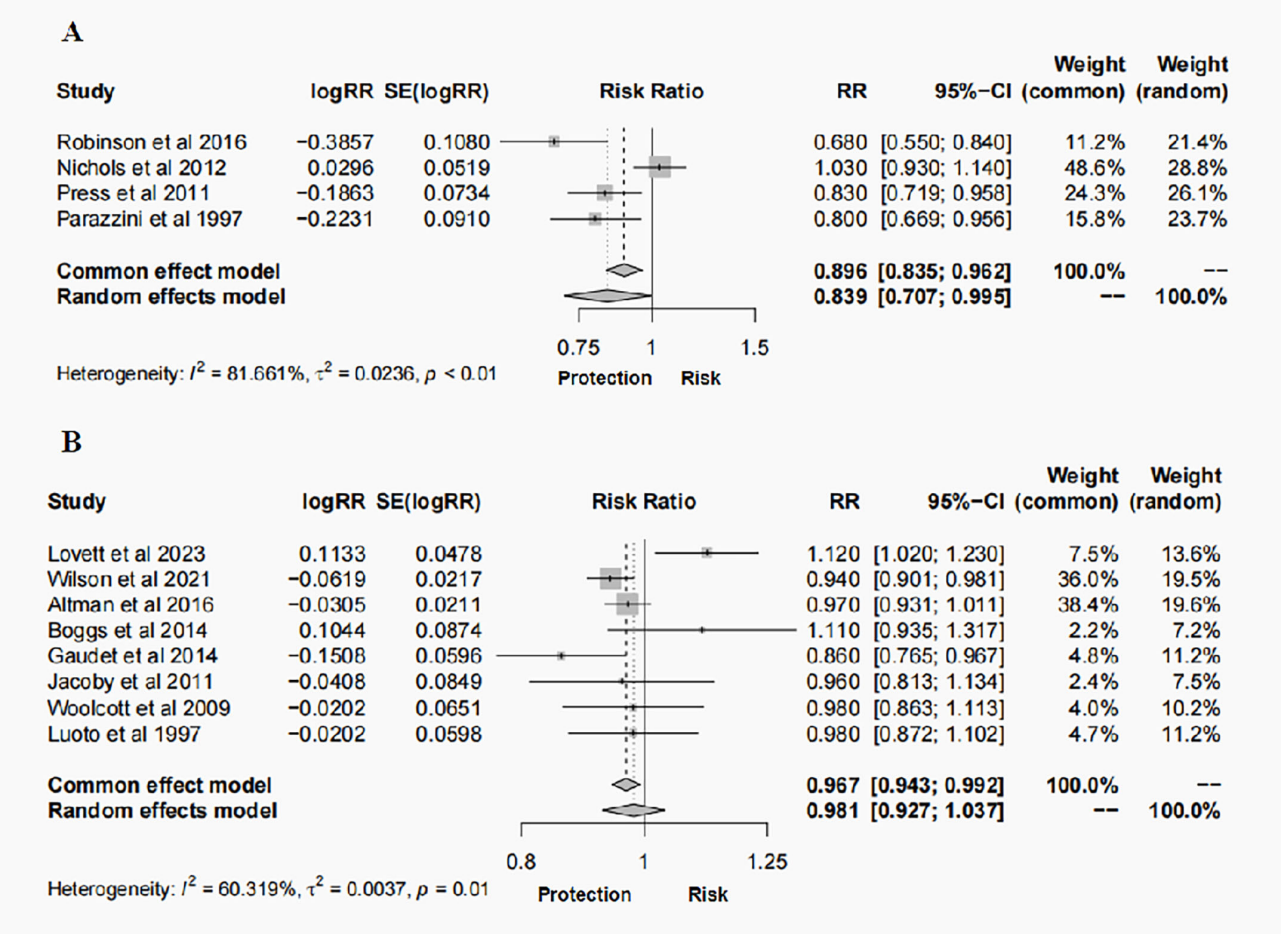
Forest plots of outcomes for the risk of BC following hysterectomy: **(A)** case–control studies; **(B)** cohort studies.

A total of 12 studies, 4 case-control studies and 8 cohort studies, reported on the risk of BC. The results of the random effects model meta-analysis showed that hysterectomy was associated with 16% reduction in risk of cancer (RR = 0.839, 95% CI: 0.707-0.995, *P* = 0.043, *I^2^* = 81.661%) (case-control studies, **Figure 2A**). However, the results of the random effects model meta-analysis showed that hysterectomy was not associated with BC (RR = 0.981, 95% CI: 0.927-1.037, *P* = 0.495, *I^2^* = 60.319%) (cohort studies, **Figure 2B**).

### 3.5 Subgroup analysis

The case-control study results showed that people with more than 10 years of follow-up after hysterectomy (RR = 0.720, 95% CI: 0.549-0.945, *I^2^* = 51.18%). When analyzed by subtype, hysterectomy was specifically associated with a reduced incidence of HR+ BC (RR = 0.739, 95% CI: 0.637-0.858, *I²* = 0.00%),which was consistent with the overall summary analysis. However, no association was observed between hysterectomy and the incidence of HR- BC. The results of subgroup analysis are presented in **Table 2**.

**Table 2:** Subgroup analyses case-control studies for the risk of BC following hysterectomy.

Subgroups	Case-control studies	RR(95% CI)	Heterogeneity	
			*P*-values	*I ^2^* (%)
Hormone therapy				
Yes	2	0.858(0.379, 1.944)	0.01	94.05
No	2	0.922(0.690, 1.231)	0.03	77.85
Follow-up				
10–19 years	2	0.720(0.549, 0.945)	0.15	51.18
≥20 years	2	0.752(0.632, 0.894)	0.64	0.00
Type				
HR+	2	0.739(0.637, 0.858)	0.48	0.00
HR-	2	0.803(0.065, 1.065)	0.15	52.64

The cohort study results showed a lower risk of BC in those undergoing hysterectomy before the age of 45 (RR = 0.945, 95% CI: 0.906-0.986, *I^2^* = 0.00%), while subgroup analyses of ethnicity showed a higher risk of BC after hysterectomy in whites (RR = 1.125, 95% CI: 1.109-1.241, *I^2^* = 0.00%). No association was observed between the risk of BC among people who underwent surgery after age 45, black people, different geographical areas, and no postoperative hormone therapy. The results of subgroup analysis are presented in **Table 3** and **Supplementary Figures 1-6**.

**Table 3:** Subgroup analyses cohort studies for the risk of BC following hysterectomy.

Subgroups	Cohort studies	RR(95% CI)	Heterogeneity	
			*P*-values	*I ^2^* (%)
Age				
<45	4	0.945(0.906, 0.986)	0.93	0.00
45-55	4	0.947(0.894, 1.003)	0.30	18.71
>55	4	0.903(0.805, 1.013)	0.73	0.00
Race				
White	2	1.125(1.109, 1.241)	0.85	0.00
Black	2	1.020(0.761, 1.368)	0.05	73.45
Geographic region				
Europe	2	0.971(0.934, 1.010)	0.87	0.00
The United States	4	0.963(0.869, 1.067)	0.10	51.63
Hormone therapy				
No	2	0.919(0.622, 1.356)	0.05	74.15
Type				
HR+	2	1.015(0.923, 1.116)	0.82	0.00
HR-	2	0.986(0.803, 1.210)	0.57	0

### 3.6 Sensitivity analysis and publication bias

**Figure 3:**
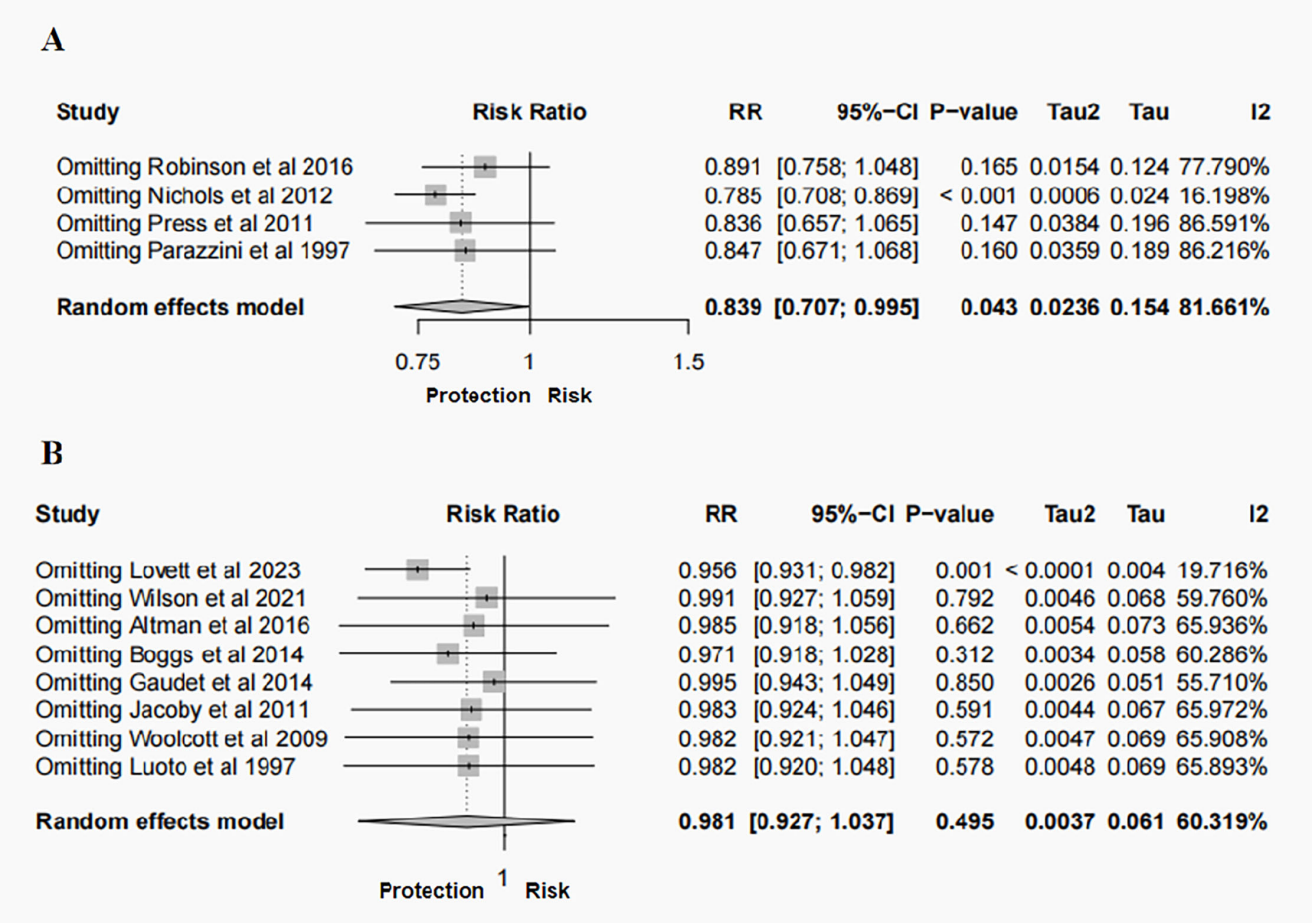
Sensitivity analysis of the meta-analysis for the risk of BC following hysterectomy: **(A)** case–control studies; **(B)** cohort studies.

**Figure 4:**
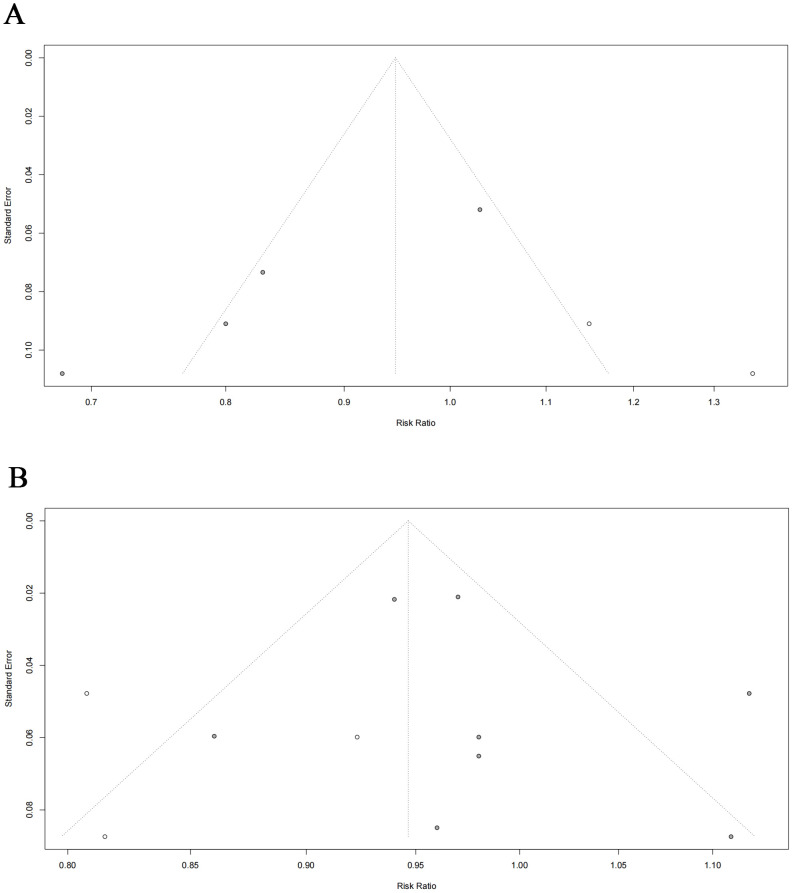
Egger’s funnel plots of the meta-analysis for the risk of BC following hysterectomy: **(A)** case–control studies ; **(B)** cohort studies.

Sensitivity analyses were performed to identify sources of heterogeneity by eliminating one study in each turn. In case-control study, when the Nichols, et al., 2012 (28) was excluded, heterogeneity was significantly reduced (RR = 0.785, 95% CI: 0.708- 0.869, *P* < 0.001, *I^2^* = 16.198%). When Lovett, et al., 2023 was excluded from the cohort study, it was observed that not only was the heterogeneity significantly reduced, but the finding that hysterectomy was not related to BC risk changed to a profound 4.4% reduction in the risk of BC after hysterectomy (RR = 0.956, 95% CI: 0.931- 0.982, *P* = 0.001, *I^2^* = 19.716%) (**Figure 3**). Notably, the narrow 95% CI (0.931-0.982) indicates a high degree of precision in this risk estimate. When other studies were removed, the initial results did not change significantly. Funnel plots and Egger’s test were performed to assess the risk of publication bias. While visual inspection of the funnel plots, particularly for cohort studies, suggested some asymmetry. However, the results of the Egger’s regression test showed no statistically significant evidence of publication bias (Case-control studies: *P* = 0.218; Cohort studies: *P* = 0.475) (**Figure 4**). And this may be due to the limited number of included studies, minor bias cannot be entirely ruled out in this meta-analysis.

## 4 Discussion

In this systematic review and meta-analysis, the meta-analysis results of case-control studies indicate that compared to those who did not undergo surgery, patients subjected to hysterectomy have a 0.16-fold reduced risk of developing BC. However, the meta-analysis of cohort studies shows no correlation between hysterectomy and BC occurrence. Both analyses exhibit significant heterogeneity, particularly in the cohort studies. Sensitivity analysis reveals that after excluding the Lovett et al., 2023 study (38), not only does the heterogeneity decrease significantly, but the finding of no association between hysterectomy and BC occurrence is reversed, indicating a notable reduction in BC risk among those who underwent hysterectomy. The profound impact of this study stems from the combination of its unique population characteristics and methodological rigor, which collectively untangle a key confounding factor potentially obscured in other studies. First, the study is based on the “Sister Study” cohort, in which all participants had a sister with BC, indicating that this population inherently possesses a higher baseline risk related to genetic factors. More importantly, through detailed stratified analyses, the authors demonstrated that hysterectomy alone was only weakly associated with BC risk (HR = 1.08, 95% CI: 0.94-1.23). In contrast, the exposure combination that significantly increased risk was “hysterectomy plus estrogen-progestin therapy” (HR = 1.25, 95% CI: 1.01-1.55). By comparison, most of the other cohort studies included in the present meta-analysis (with the exception of the study by Jacoby et al., 2011) failed to adequately control for this critical confounder—postoperative combined hormone therapy. Consequently, the additional risk attributable to combined therapy in those studies may have been erroneously attributed to hysterectomy itself, leading to an overestimation of risk in the “hysterectomy-only” group. This sensitivity analysis does not merely involve excluding one large study; rather, it removes a major source of confounding, thereby clarifying the origin of heterogeneity. Furthermore, the sensitivity analysis of the cohort studies not only untangled a significant protective association but also provided a precise estimate of the effect, as evidenced by the narrow confidence interval. Our subgroup analysis yields interesting findings, albeit with a small sample size and less reliability compared to the primary analysis. Subgroup analysis based on case-control studies shows a significant negative correlation between hysterectomy and BC risk in populations with more than 10 years of follow-up and in BC patients with a post-disease pathological diagnosis of HR +. Meanwhile, subgroup analysis based on cohort studies suggests that those who underwent hysterectomy before the age of 45 have a lower risk of BC, while white individuals show a 1.13-fold increased risk of BC after hysterectomy.

Regarding the reduced risk of BC among patients who have undergone simple hysterectomy, we hypothesize that this may be influenced by both anatomical and endocrine pathways. Firstly, although simple hysterectomy preserves the ovaries, the disruption of utero-ovarian vascular anastomoses during surgery can lead to a 50%-70% reduction in ovarian blood flow, inducing ischemic ovarian failure (39, 40). The pathological features include a decrease in antral follicle count (AFC) and anti-Mullerian hormone (AMH) levels within six months postoperatively, which can also be evidenced by various menopausal symptoms, bone loss, and increased risk of hypertension (41–43). Meanwhile, the uterus, as a crucial endocrine organ, its removal results in the sudden loss of uterine-derived regulatory factors (such as prostaglandin F2α and relaxin), which may alter the negative feedback mechanism of the hypothalamus-pituitary-ovarian axis, leading to elevated serum follicle-stimulating hormone (FSH) and luteinizing hormone (LH) levels postoperatively (44). Studies have shown that these changes collectively advance the average natural menopause age by 3.7 years compared to the non-surgical population (39). Epidemiological models indicate that for every one-year advance in menopause age, the relative risk of BC decreases by 2.3% (45, 46). Therefore, an early menopause of 3.7 years may imply a 7% to 11% risk reduction. This aligns with our subgroup analysis findings, where women under 45 years old who underwent surgery had a reduced risk of BC. Postoperative endocrine changes include increased estradiol fluctuation, decreased estrone/estradiol ratio, and downregulation of ERα expression and aromatase activity, which may confer protection (47). Notably, preoperative medical treatments may enhance this protective effect. For instance, patients with endometriosis receiving GnRH agonist therapy for ≥6 months can reduce their risk by 31% (48). Secondly, over 90% of hysterectomies are performed for benign indications, including uterine fibroids (leiomyomas), endometriosis, uterine prolapse, and menstrual disorders (49). However, some risk factors for these diseases contradict those for BC. For example, alcohol consumption and body mass index (BMI) can reduce the risk of endometriosis but increase the risk of postmenopausal BC (50–52). Childbirth and early first pregnancy can elevate the risk of prolapse but lower the risk of BC (53–55). This suggests that due to these conflicting risk factors, hysterectomy may reduce the risk of BC.

The finding of no correlation between hysterectomy and BC risk may stem from preoperative and postoperative interventions. These interventions may produce a biological neutralizing effect. Since women may have already received medical treatment for hysterectomy indications before surgery, and these treatments potentially influence BC risk. For instance, treatments for endometriosis include danazol, oral contraceptives, and growth hormone-releasing hormone agonists (56–58). The preoperative hormone therapy may balance the hormonal changes postoperatively, forming a biological neutralization mechanism, which could lead to the observed zero association between hysterectomy and BC risk. It’s worth noting that the pathophysiological characteristics of the surgical indication disease itself may produce reverse regulation: endometrial cells in patients with endometriosis show a 2.3-fold upregulation of BRCA1 expression, and this enhanced DNA repair capability may partially offset the carcinogenic effects of estrogen exposure; while abnormal pelvic floor collagen metabolism in patients with uterine prolapse may inhibit breast tumor microenvironment formation by altering stroma-epithelial interactions (59).

An interesting finding from our subgroup analysis is that the risk of BC among white women increases by 1.23 times after hysterectomy. Previous studies have indicated that the incidence of simple hysterectomy varies among different races (60–62). Compared to black women, white women have a lower prevalence of hysterectomy for benign diseases, a higher average age at surgery, and a higher proportion of hormone therapy use (63). We believe these differences may be due to racial disparities in the incidence and severity of uterine pathologies, early treatments to prevent hysterectomy, or medical practices (61, 64, 65). Therefore, regarding the increased risk of BC, we hypothesize that it is related to the interaction between genetic susceptibility and environmental exposure. Firstly, diseases leading to hysterectomy may share the same hormonal etiology with BC. Some risk factors for these diseases are also known as risk factors for BC, such as early menarche, low parity, BMI, and inadequate physical activity (66). Secondly, diseases like endometriosis may be associated with BC risk, although the correlation is not yet clear. However, some studies have shown that they may slightly increase the risk of BC (59, 67). Additionally, the retention of adnexa after hysterectomy may lead to the formation of pelvic fluid, and this chronic inflammatory state may promote epithelial-mesenchymal transition (EMT) by activating the NF-κB pathway, affecting cancer development (68). Finally, postoperative use of estrogen combined with progesterone therapy may also increase BC risk. Although our subgroup analysis did not observe a significant impact of postoperative hormone use on the study results, it may be related to the limited number of studies included (only two with high heterogeneity). Therefore, more high-quality studies are needed in the future to further validate these findings.

The primary source of heterogeneity in our cohort study was the 2023 study by Lovett et al (38). We analyzed possible reasons for this heterogeneity. One reason may be the inclusion of patients with a family history of BC, which may increase their risk of developing the disease. Another reason could be potential measurement errors in hysterectomy status, as self-reporting and recall may introduce inaccuracies. Future research should construct multi-dimensional predictive models that integrate surgical parameters, dynamic hormone monitoring, and genomic features. Simultaneously, multi-omics longitudinal studies covering the epigenome, metabolome, and immune microenvironment (such as dynamic monitoring from preoperative to 3 and 10 years postoperatively) should be conducted. In clinical practice, it is recommended to establish an individualized BC risk assessment system for patients under 45 years old undergoing hysterectomy. This system should incorporate genetic risk scores, and postoperative hormone replacement therapy plans into the decision-making process and develop targeted monitoring programs for high-risk populations. These measures will help unravel the precise dose-effect relationship between hysterectomy and BC risk and provide evidence-based medical support for the cancer prevention value of gynecological surgery.

Prior to the initiation of this study, existing research had investigated the impact of hysterectomy combined with bilateral salpingo-oophorectomy on cancer risk and mortality through meta-analysis (23). However, the uniqueness of the present study lies in the fact that it is the first to specifically examine the association between hysterectomy performed solely for benign indications and the risk of BC. It separately analyzes the included case-control studies and cohort studies and conducts an in-depth subgroup analysis to explore the influence of factors such as age, ethnicity, follow-up time, and hormone therapy. Notably, it reveals a significant reduction in risk for young women (<45 years old) and patients with HR+ BC.

However, this study also has some limitations. Firstly, the contradictory results between case-control and cohort studies may stem from methodological differences. The high heterogeneity observed within each study type suggests the potential presence of unmeasured confounding factors. Second, most studies did not document medication history or provide risk estimates stratified by surgical indications, which may have led to observed associations being attributable to underlying diseases or medication use rather than to the surgery itself. Additionally, the geographical and ethnic distribution of the included studies is uneven, with a predominance of research based in the United States, which may limit the generalizability of the results. Finally, some subgroups have small sample sizes, such as those involving two studies on hormone therapy, which results in insufficient statistical power and higher uncertainty in the findings. Future prospective studies should collect more detailed data on surgical indications, medication history, and other relevant factors, as well as include more diverse populations.

## 5 Conclusion

In summary, the study demonstrated a potential association between hysterectomy and BC risk, especially for premenopausal women. However, given the limitations of the available data, future studies are needed to further validate these factors, including the potential biases introduced using hormones after hysterectomy and whether preoperative disease is analyzed as an exposure factor in BC risk investigations.

## Data Availability

The original contributions presented in the study are included in the article/**Supplementary Material**. Further inquiries can be directed to the corresponding author.
