# Supplementary material for: Association between hysterectomy for benign indications and the risk of breast cancer: a systematic review and meta- analysis
Source: Front Oncol. 2025 Nov 20;15:1600459. doi: 10.3389/fonc.2025.1600459 (PMC12675176; doi:10.3389/fonc.2025.1600459)
Supplement: Supplementary file 1 [file DataSheet1.docx]

Supplementary Material

# Supplementary Data

**Supplementary Tables**

Supplementary Table 1 - Search strategy for PubMed

Supplementary Table 2 - Search strategy for Cochrane Library

Supplementary Table 3 - Search strategy for Embase

Supplementary Table 4 - Further Characteristics of the included studies

Supplementary Table 5 - Quality assessment of included case-control studies by Newcastle-Ottawa Scale

Supplementary Table 6 - Quality assessment of included cohort studies by Newcastle-Ottawa Scale

**Supplementary Figures**

Supplementary Figure 1- Forest plot of subgroup analysis (age)

Supplementary Figure 2 - Forest plot of subgroup analysis (follow-up)

Supplementary Figure 3 -Forest plot of subgroup analysis (hormone therapy)

Supplementary Figure 4 -Forest plot of subgroup analysis (location)

Supplementary Figure 5 -Forest plot of subgroup analysis (race)

Supplementary Figure 6 -Forest plot of subgroup analysis (type)

# Supplementary Figures and Tables

## Supplementary Tables

## Supplementary Tables 1~3: Electronic Search Criteria for Systematic Review of Literature.

(1) PubMed (November 11, 2024)

| Database | Search criteria |
| --- | --- |
| Pubmed | #1 ((((((((((((((((((((((((((((((((((((("Breast Neoplasms"[Mesh]) OR (Breast Neoplasm[Title/Abstract])) OR (Neoplasm, Breast[Title/Abstract])) OR (Breast Tumors[Title/Abstract])) OR (Breast Tumor[Title/Abstract])) OR (Tumor, Breast[Title/Abstract])) OR (Tumors, Breast[Title/Abstract])) OR (Neoplasms, Breast[Title/Abstract])) OR (Breast Cancer[Title/Abstract])) OR (Cancer, Breast[Title/Abstract])) OR (Mammary Cancer[Title/Abstract])) OR (Cancer, Mammary[Title/Abstract])) OR (Cancers, Mammary[Title/Abstract])) OR (Mammary Cancers[Title/Abstract])) OR (Malignant Neoplasm of Breast[Title/Abstract])) OR (Breast Malignant Neoplasm[Title/Abstract])) OR (Breast Malignant Neoplasms[Title/Abstract])) OR (Malignant Tumor of Breast[Title/Abstract])) OR (Breast Malignant Tumor[Title/Abstract])) OR (Breast Malignant Tumors[Title/Abstract])) OR (Cancer of Breast[Title/Abstract])) OR (Cancer of the Breast[Title/Abstract])) OR (Mammary Carcinoma, Human[Title/Abstract])) OR (Carcinoma, Human Mammary[Title/Abstract])) OR (Carcinomas, Human Mammary[Title/Abstract])) OR (Human Mammary Carcinomas[Title/Abstract])) OR (Mammary Carcinomas, Human[Title/Abstract])) OR (Human Mammary Carcinoma[Title/Abstract])) OR (Mammary Neoplasms, Human[Title/Abstract])) OR (Human Mammary Neoplasm[Title/Abstract])) OR (Human Mammary Neoplasms[Title/Abstract])) OR (Neoplasm, Human Mammary[Title/Abstract])) OR (Neoplasms, Human Mammary[Title/Abstract])) OR (Mammary Neoplasm, Human[Title/Abstract])) OR (Breast Carcinoma[Title/Abstract])) OR (Breast Carcinomas[Title/Abstract])) OR (Carcinoma, Breast[Title/Abstract])) OR (Carcinomas, Breast[Title/Abstract]) |
|  | #2 (hysterectomy [MeSH Terms] OR (hysterectomy* [Title/Abstract] ) ) |
|  | #3 (risk[Title/Abstract] OR risk[MeSH:noexp] OR mortality[Title/Abstract] OR mortality[MeSH:noexp] OR cohort[Title/Abstract]) |
|  | #4 #1 AND #2 AND #3 |

(2) Cochrane Library(November 11, 2024)

| Database | Search criteria |
| --- | --- |
| Cochrane Library | #1 MeSH descriptor: [Breast Neoplasms] explode all trees |
|  | #2 (breast cancer*):ti,ab,kw |
|  | #3 (breast neoplasm*):ti,ab,kw |
|  | #4 (breast carcinoma*):ti,ab,kw |
|  | #5 (breast tumour*):ti,ab,kw |
|  | #6 (breast tumor*):ti,ab,kw |
|  | #7 #1 OR #2 OR #3 OR #4 OR #5 OR #6 |
|  | #8 (hysterectomy):ti,ab,kw (Word variations have been searched) |
|  | #9 #7 AND #8 |

(3) Embase (November 11, 2024)

| Database | Search criteria |
| --- | --- |
| Embase | #1 ('breast':ab,ti OR 'mammary':ab,ti) AND ('cancer':ab,ti OR 'neoplasm':ab,ti OR 'oncology':ab,ti OR 'tumor':ab,ti OR 'malignancy':ab,ti OR 'carcinoma':ab,ti OR 'adenocarcinoma':ab,ti OR 'sarcoma':ab,ti) |
|  | #2 'hysterectomy'/exp OR hysterectomy:ti,ab,kw |
|  | #3 'risk':ab,ti OR 'mortality':ab,ti OR 'cohort':ab,ti |
|  | #4 #1 AND #2 AND #3 |

**Supplementary Table 4**: Further Characteristics of the included studies

| **Author** | **Year** | **Design** | RR（95% CI） | OR（95% CI） | HR（95% CI） |
| --- | --- | --- | --- | --- | --- |
| Lovett et al | 2023 | Prospective cohort | / | / | 1.12(1.02-1.23) |
| Wilson et al | 2021 | Retrospective cohort study | / | / | 0.94(0.90-0.98) |
| Altman et al | 2016 | Prospective cohort | / | / | 0.68(0.55-0.84) |
| Robinson et al | 2016 | Case-control study | 0.68(0.55-0.84) | / | / |
| Gaudet et al | 2014 | Prospective cohort | 0.86(0.76-0.96) | / | / |
| Boggs et al | 2014 | Prospective cohort | / | / | 1.11(0.93-1.31) |
| Nichols et al | 2012 | Case-control study | / | 1.03(0.93-1.14) | / |
| Press et al | 2011 | Case-control study | / | 0.83(0.72-0.96) | / |
| Jacoby et al | 2011 | Prospective cohort | / | / | 0.96(0.81-1.13) |
| Woolcott et al | 2009 | Prospective cohort | 0.98（0.86-1.11） | / | / |
| Parazzini et al | 1997 | Case-control study |  | 0.8(0.7-1.0) | / |
| Luoto et al | 1997 | Retrospective cohort study | 0.98（0.87-1.10） | / | / |

Abbreviations: RR:Risk Ratio; HR: Hazard Ratio; OR: Odds Ratio; 95%CI: 95% confidence intervals.

**Supplementary Table 5**: Quality assessment of included case-control studies by Newcastle-Ottawa Scale.

| Author, year | Selection | | | |  | Comparability | |  | Exposure | | | Score |
| --- | --- | --- | --- | --- | --- | --- | --- | --- | --- | --- | --- | --- |
|  | Adequacy of the case definition | Representativeness of cases | Choice of controls | Definition of control |  | Study controls for age | Study controls for any additional important factor |  | Exposure assessment | The method of exposure assessment | Non-response rate |  |
| Robinson et al, 2016 | ★ | ★ | ★ | ★ |  | ★ | ★ |  |  | ★ |  | 7 |
| Nichols et al, 2012 | ★ | ★ | ★ | ★ |  | ★ | ★ |  |  | ★ |  | 7 |
| Press et al, 2011 | ★ | ★ | ★ | ★ |  | ★ | ★ |  | ★ | ★ | ★ | 9 |
| Parazziniet al, 1997 | ★ | ★ | ★ | ★ |  | ★ | ★ |  | ★ | ★ | ★ | 9 |

Case-control Studies: **Selection** ① Adequacy of the case definition★ ②Representativeness of cases★ ③Choice of controls★ ④Definition of control★. **Comparability** ① Comparability of case-controls on the basis of the design or analysis★★. **Outcome** ① Exposure assessment★ ② The method of exposure assessment★ ③ Non-response rate★.

**Supplementary Table 6** - Quality assessment of included cohort studies by Newcastle-Ottawa Scale

| Author, year | Selection | | | |  | Comparability | |  | Outcome | | | Score |
| --- | --- | --- | --- | --- | --- | --- | --- | --- | --- | --- | --- | --- |
|  | Representativeness of exposed cohort | Selection of non-exposed cohort | Exposure Ascertainment | Outcome present at start of study |  | Study controls for age | Study controls for any additional important factor |  | Assessment of Outcome | Length of follow-up | Adequacy of follow-up |  |
| Lovett et al, 2023 | ★ | ★ |  | ★ |  |  | ★ |  |  | ★ | ★ | 6 |
| Wilson et al, 2021 | ★ | ★ | ★ | ★ |  | ★ | ★ |  | ★ | ★ | ★ | 9 |
| Altman et al, 2016 | ★ | ★ | ★ | ★ |  | ★ |  |  | ★ |  | ★ | 7 |
| Gaudet et al, 2014 | ★ | ★ | ★ | ★ |  | ★ | ★ |  | ★ |  | ★ | 8 |
| Boggs et al, 2014 | ★ | ★ |  | ★ |  | ★ | ★ |  | ★ |  | ★ | 7 |
| Jacoby et al, 2011 | ★ | ★ |  | ★ |  | ★ | ★ |  |  |  | ★ | 6 |
| Woolcott et al, 2009 | ★ | ★ |  | ★ |  | ★ | ★ |  |  |  | ★ | 6 |
| Luoto et al, 1997 | ★ | ★ |  | ★ |  |  |  |  | ★ | ★ | ★ | 6 |

Cohort Studies: **Selection** ① Representativeness of the exposed cohort★ Selection of the non exposed cohort★ ② Ascertainment of exposure★ ③ Demonstration that outcome of interest was not present at start of study★. **Comparability** ① Comparability of cohorts on the basis of the design or analysis★★. **Outcome** ① Assessment of outcome★ ② Was follow-up long enough for outcomes to occur★ ③ Adequacy of follow up of cohorts★.

## 2.2 Supplementary Figures

##
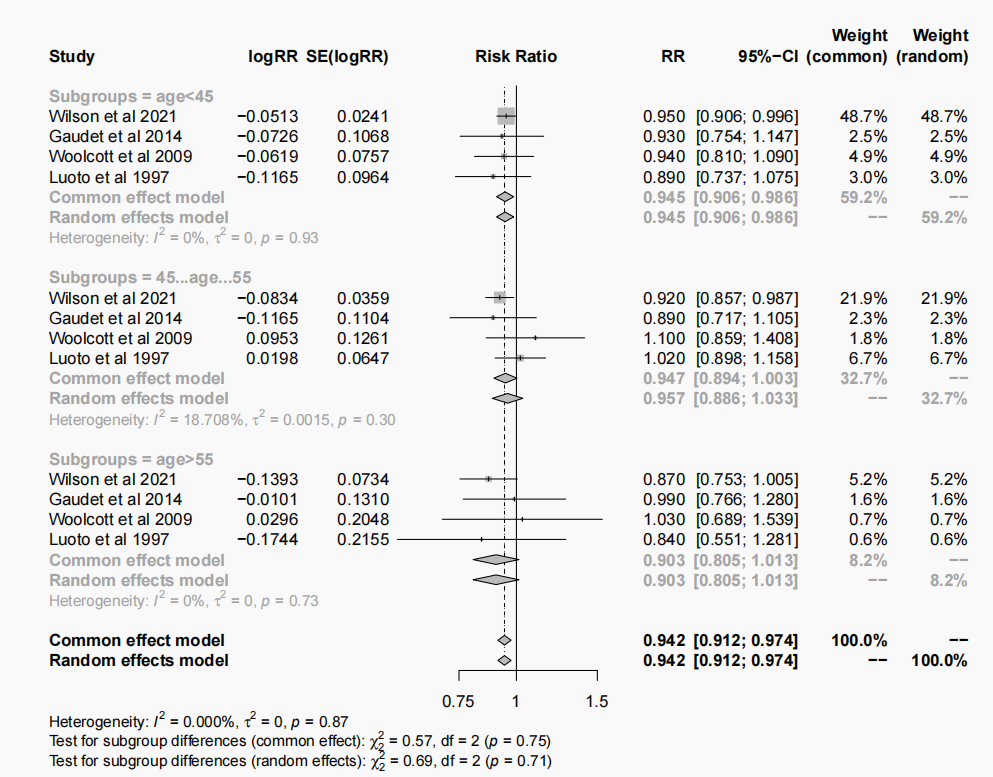


## Supplementary Figure 1. Forest plot of subgroup analysis (age)


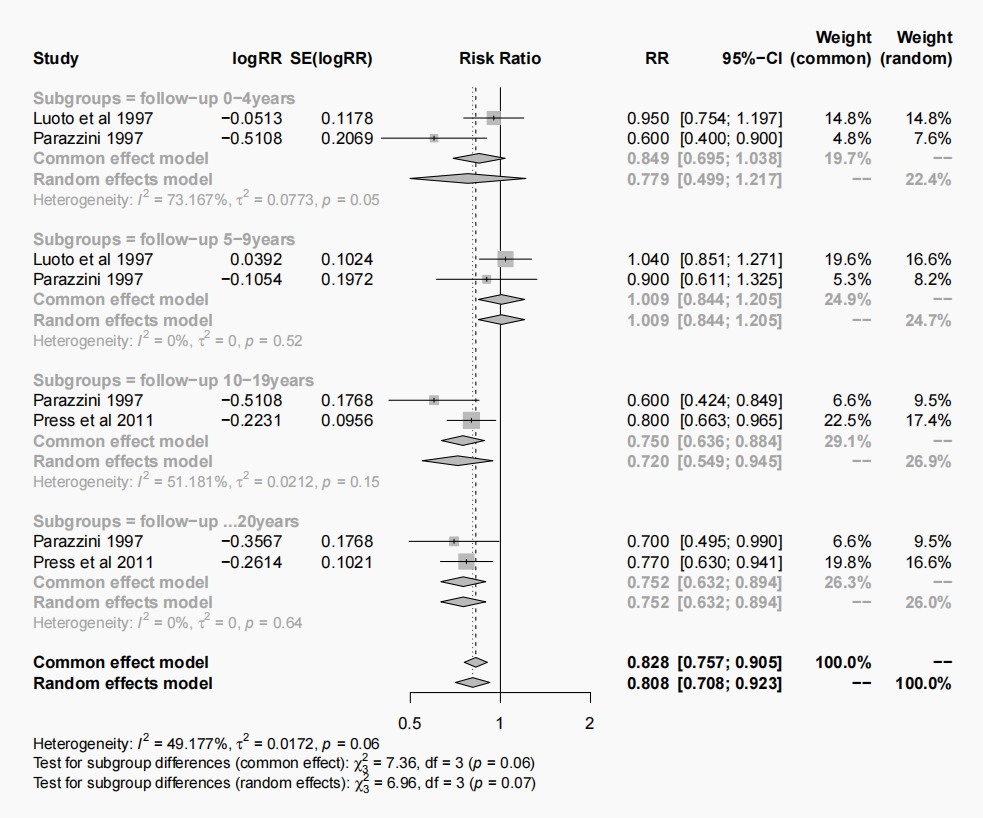


**Supplementary Figure 2.** Forest plot of subgroup analysis (follow-up)


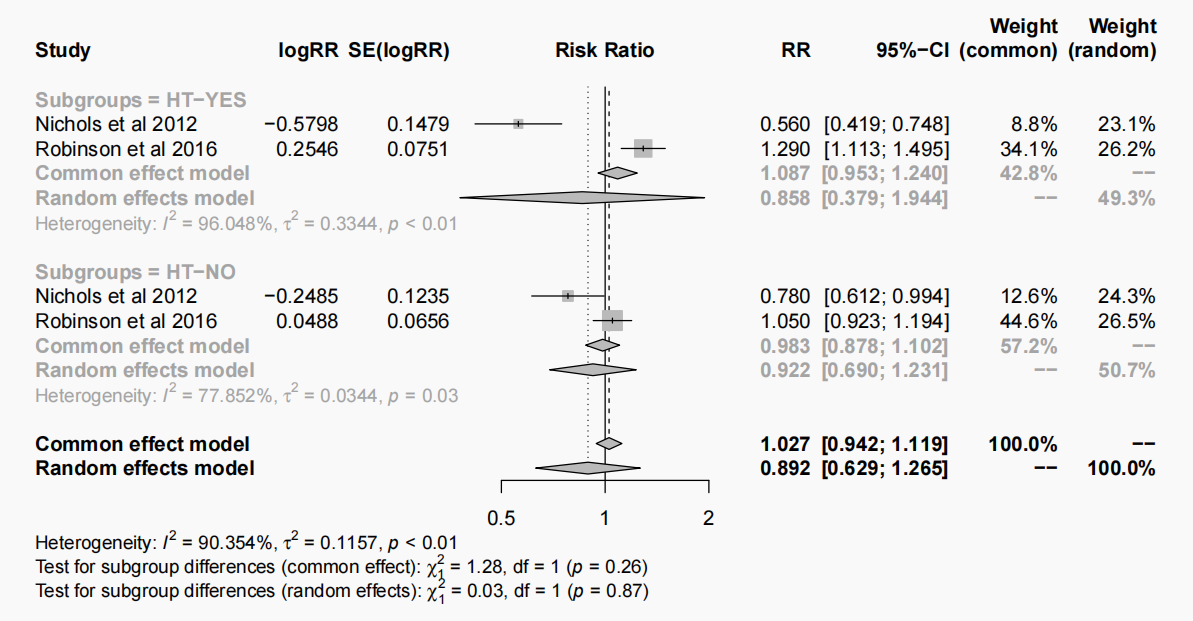


(A) Case-control studies


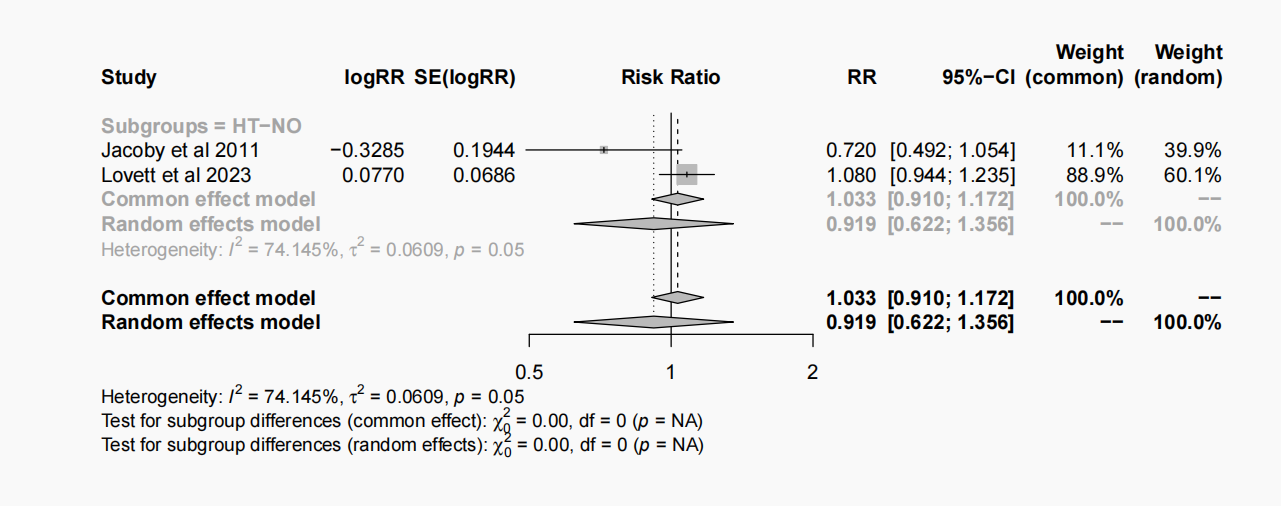


(B) Cohort studies

**Supplementary Figure 3.** Forest plot of subgroup analysis (hormone therapy)


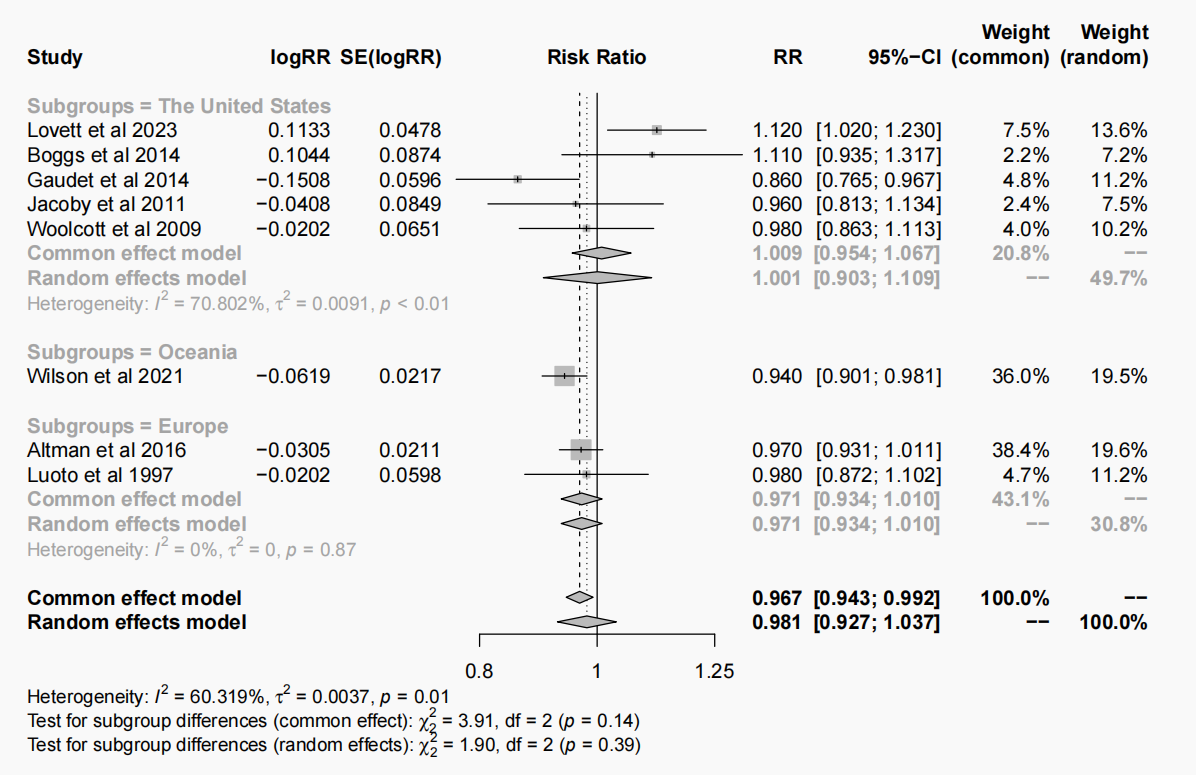


**Supplementary Figure 4.** Forest plot of subgroup analysis (location)


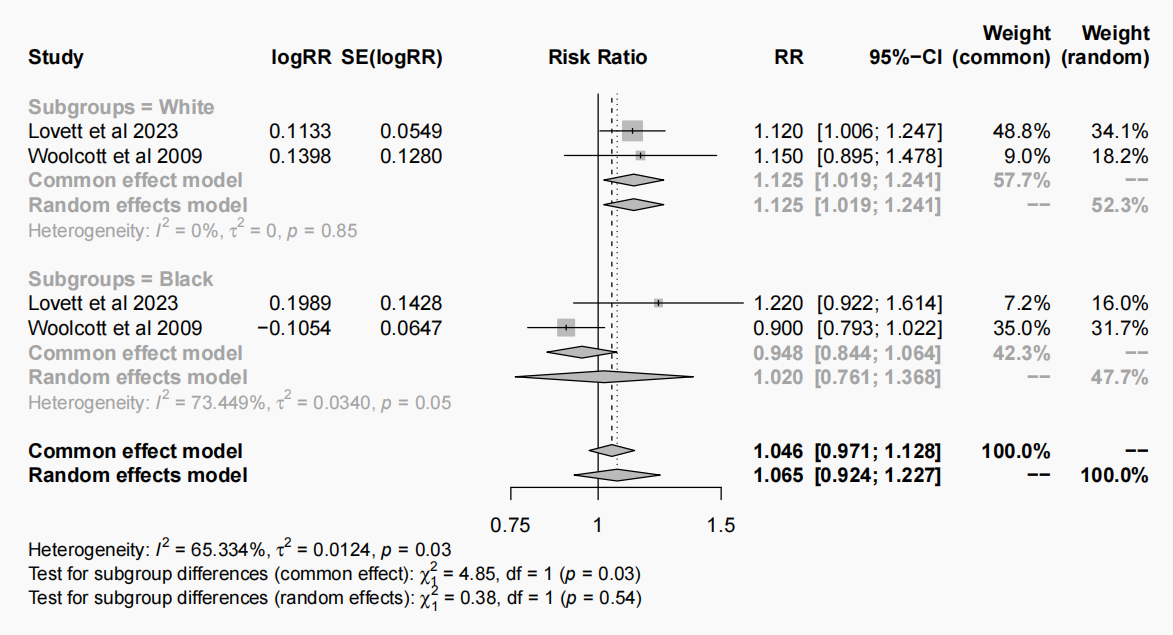


**Supplementary Figure 5**. Forest plot of subgroup analysis (race)


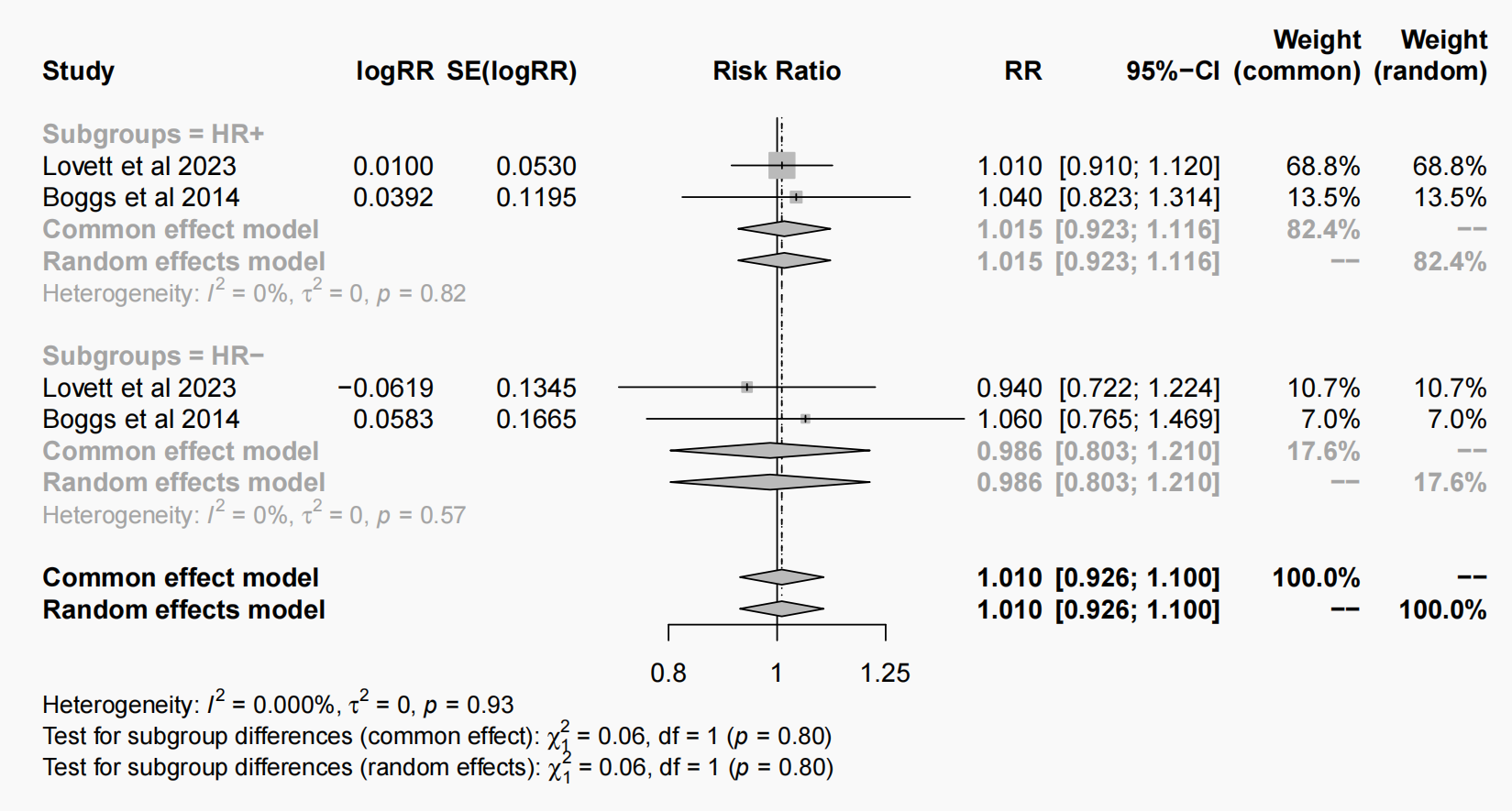


**Supplementary Figure 6.** Forest plot of subgroup analysis (type)
